# Quarter Century Management of Chronic Ureteropelvic Junction Obstruction in a Solitary Kidney with a Ureteral Stent

**DOI:** 10.1089/cren.2017.0144

**Published:** 2018-04-01

**Authors:** Garen Abedi, Roshan M. Patel, Cyrus Lin, Ralph V. Clayman

**Affiliations:** Department of Urology, University of California, Irvine, Irvine, California.

**Keywords:** ureteral stent, ureteropelvic junction obstruction, solitary kidney, renal insufficiency

## Abstract

***Background:*** The ureteral stent provides a conduit for urinary drainage from the kidney to the bladder and is integral to contemporary urologic practice. A ureteral stent is often utilized in acute conditions to prevent or overcome obstruction; however, in nonsurgical patients, because of disease or preference, a ureteral stent may be used as a last resort for long-term management of a stricture in lieu of a nephrostomy tube. This case highlights a patient whose chronic ureteral obstruction has been managed with an indwelling ureteral stent for 25 years; remarkably, stent exchanges are currently required only every 2 years.

***Case Presentation:*** A 33-year-old man initially presented with a solitary left kidney and a ureteropelvic junction obstruction. The patient's right kidney was nonfunctioning since childhood because of a presumed ureteropelvic junction obstruction with grade IV hydronephrosis. The patient underwent two failed open repairs of the left kidney in the 1980s, resulting in a totally intrarenal, constricted renal pelvis; an endopyelotomy in 1992 also failed and required angioembolizaton of a segmental renal vessel. The patient refused any further surgical procedures and thus has been managed exclusively with a 7/14F × 28 cm endopyelotomy stent (Boston Scientific^®^) for 25 years; the interval between stent changes was slowly expanded until they are now being done at 2-year intervals. The patient has not developed recurrent urinary tract infections, stent colic, or stent encrustation.

***Conclusion:*** Patients who require chronic indwelling ureteral stents are rare. In this situation, with careful monitoring, the interval between stent exchanges was extended to 2 years, thereby precluding a chronic nephrostomy tube.

## Introduction

Treatment of benign ureteropelvic junction (UPJ) obstruction consists of either surgical reconstruction in the form of pyeloplasty or endoscopic management with endopyelotomy. Patients who fail both modes of therapy and refuse further surgery can opt for a chronic indwelling percutaneous nephrostomy, a nephroureteral stent (e.g., not available in the United States), or undergo chronic ureteral stent exchanges.^1^

Ureteral stents have undergone numerous iterations since their initial description. For example, the development of the metal ureteral stents (e.g., Resonance stent—Cook Medical^®^) has allowed longer span of time between stent changes for patients with chronic ureteral obstruction.^2^

However, one of the most concerning complications of long-term ureteral stents is encrustation, which can potentially lead to obstruction and subsequent permanent renal dysfunction as well as urosepsis. The longer a stent is left in place, the higher chance there is for encrustation.^3^ Depending on patient characteristics and stent material, the recommended time for exchange of chronic ureteral stents has ranged from 3 months for most nonmetal-based stents to 12 months for the metal stents. However, to the best of our knowledge, a routine planned period of 2 years between ureteral stent exchanges has previously not been reported in the literature. This case illustrates a rare scenario wherein despite a period of 25 years of stent changes for chronic UPJ obstruction, no stent complications have been encountered while renal function has been preserved.

## Presentation of Case

**FIG. 1.**
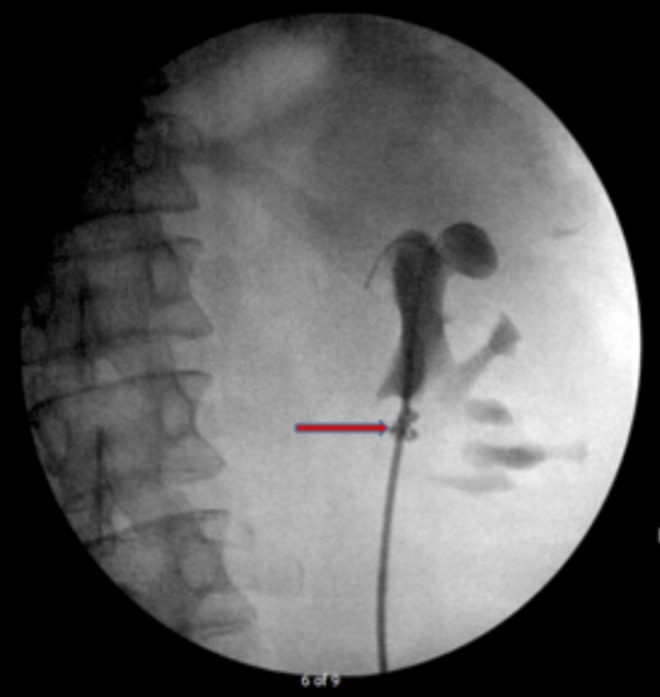
Retrograde pyelogram in 2016, during stent exchange. The guidewire is coiled in the dilated upper pole calix—this is where the proximal stent coil continues to be placed. Note the severely contracted renal pelvis. An embolization coil (*arrow*) is present at the site of the failed 1992 endopyelotomy.

A 33-year-old man with history of chronic left UPJ obstruction presented to our urology department in 1992 after having failed two open dismembered pyeloplasties. At this time, all that remained was a small sliver of renal pelvis that was completely intrarenal. In addition, he had a functionally solitary left kidney presumably secondary to UPJ obstruction with subsequent grade IV hydronephrosis and parenchymal atrophy. In 1992, we attempted a left endopyelotomy with placement of a free urothelial graft; however, the procedure was complicated by injury to a segmental renal artery, leading to angiography and embolization. A 7/14F endopyelotomy stent was left in place; his creatinine rose to 2.3 mg/dL. Despite extensive discussions regarding alternative therapies, the patient opted to proceed with chronic indwelling stent changes for management of his UPJ obstruction. In 2006, on CT imaging that was obtained at an overseas facility for routine follow-up, we noted multiple small (<3 mm) stones in the lower pole. These were basket extracted from the kidney at the time of stent change. During ureteroscopy, it was noted that part of the endovascular coil that had been deployed previously had eroded into the collecting system along the medial aspect of the UPJ (Fig. 1). At this time, interventional radiology was consulted intraoperatively and a decision was made to avoid any manipulation and continue with standard stent exchange. There were no encrustations of the exposed endovascular coil noted at the time of ureteroscopy.

**FIG. 2.**
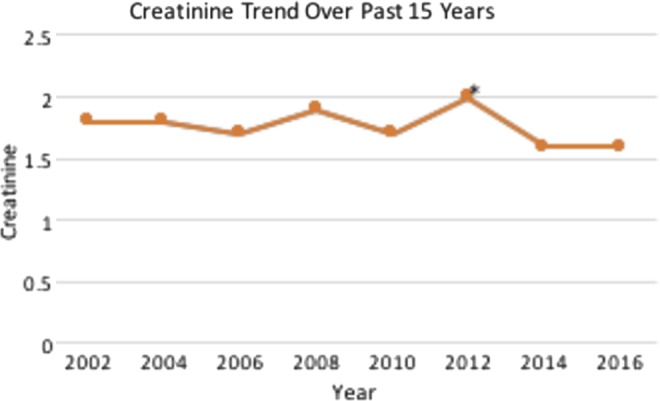
Patient's creatinine trend for the past 15 years. *Creatinine at the time of right nephrectomy.

Stent exchanges, in sequence, were performed at intervals of 4, 5, 8, 12, 14, 18, and then 25 months without noticeable stent encrustation. Furthermore, his renal function has remained stable with creatinine ranging from 1.6 to 2.0 for the past 15 years (Fig. 2). Of note, in 2012, his hypertension had become increasingly difficult to control despite multiple antihypertensive medications, including losartan, clonidine, amlodipine, and hydrochlorothiazide. Thus, given the poor function of the right kidney and the possibility it was contributing to his hypertension, he underwent a right laparoscopic nephrectomy at our institution; no renin levels were obtained because of the patient's refusal. After his nephrectomy, his hypertension is under better control on only a three-drug regimen of losartan, clonidine, and amlodipine; at his most recent visit in 2016, his blood pressure was 146/80.

## Discussion and Literature Review

Contemporary ureteral stents are used in the setting of both acute and chronic ureteral obstruction. In the latter circumstance, lengthier duration of time between stent changes may result in infection and stent encrustation.^4^ As such, the recommended stent exchange interval is from 3 to 6 months for nonmetallic stents.^4^ Alternative options for treatment of ureteral obstruction that may allow a longer indwelling time include segmental metal stents and the Detour subcutaneous bypass stent (Coloplast, Peterborough, UK).^5,6^ The latter option is currently not approved for use in the United States. The limit of how long one can wait before exchanging a ureteral stent is untested. The required timing is highly individualized as noted by Park et al..^7^ Among nine patients with “forgotten” indwelling ureteral stents, after an average of 36 months, six patients had only minimal encrustation. Assessment of the urine of these six patients revealed a low specific gravity (<1.005), indicative of high fluid intake or renal insufficiency. The remaining three patients who had extensive stent encrustation had specific gravity of >1.012. To the best of our ability, we could find only one other published instance of a forgotten stent with minimal encrustation at 40 months at the time of stent removal.^8^ Of note, this patient had pre-existing moderate renal dysfunction leading to poor concentrating ability, whereas our patient had a urine specific gravity of 1.015 in 2016.

The presented case, to our best knowledge, represents the longest duration at 25 years of management of chronic ureteral obstruction with an indwelling ureteral stent with a planned change interval of 2 years. Renal function has been stable throughout this period and there have been no stent-specific complications. It is to be noted that the stent used in the presented case was an endopyelotomy stent that has no side ports in the shaft of the stent unlike the traditional Double-J ureteral stent. In addition, the endopyelotomy stent is made from Percuflex material (Medi-tech, Watertown, MA), which may have a different encrustation rate compared with other materials.^9^ Whether using an endopyelotomy stent as opposed to a traditional Double–J ureteral stent lengthens the time between routine changes has yet to be determined. Based on manufacturer recommendations, stents should be changed at 3- to 12-month intervals depending on the type of stent material; the longer interval is specified only for metal stents. However, in the patient with a chronic indwelling stent, the timing of subsequent stent exchanges can be adjusted based on the amount of encrustation at the time of stent exchange. This was done in the present case as stent intervals were slowly increased: 4, 5, 8, 12, 14, 18, and then 25 months. It is possible that urine specific gravity may be used for predicting the potential for stent encrustation; however, this will need to be further evaluated in a prospective manner in the future and had no bearing in our case.

## Conclusion

Although the majority of chronic indwelling ureteral stents are routinely changed every 3 to 4 months, it is possible to alter this regimen based on the amount of stent encrustation of the removed stent. In this manner, stent intervals may be increased to at least 2 years. However, manufacturer recommendations for indwelling times for ureteral stents should be closely followed given limited data on prolonged change intervals.

## Abbreviations Used

CT: computed tomography
UPJ: ureteropelvic junction
